# Supplementary figures and images for: DeepCAGE: Incorporating Transcription Factors in Genome-wide Prediction of Chromatin Accessibility
Source: Genomics Proteomics Bioinformatics. 2022 Mar 12;20(3):496–507. doi: 10.1016/j.gpb.2021.08.015 (PMC9801045; doi:10.1016/j.gpb.2021.08.015)

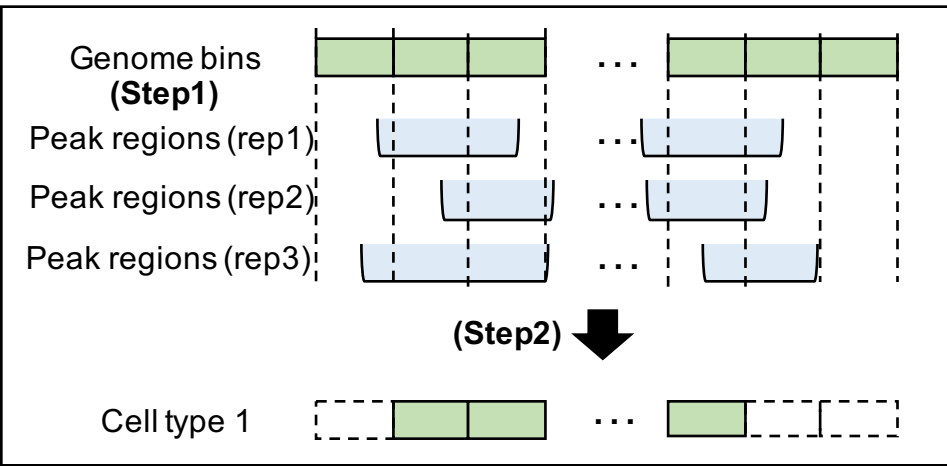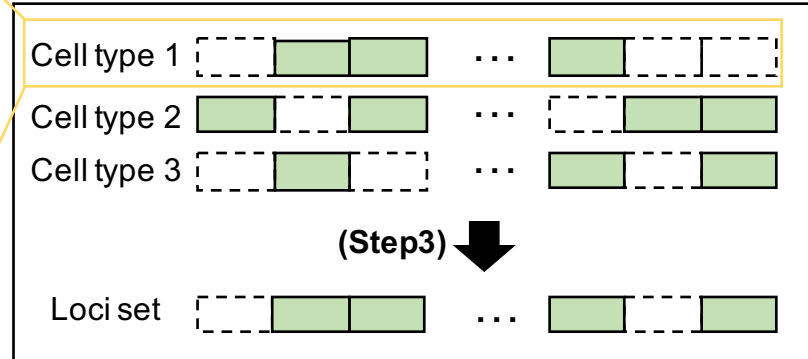

Supplement: Supplementary Figure S1 — Identification of putative accessible loci Putative accessible loci were determined by DNase-seq peaks according to the following three steps. Step 1: The human reference genome was divided into non-overlapping regions of 200 bps. Step 2: Regions is kept if it is contained in half of replicated of a cell type. Step 3: Putative accessible loci were determined by collecting regions that appear in at least two cell types. [file mmc1.pdf]

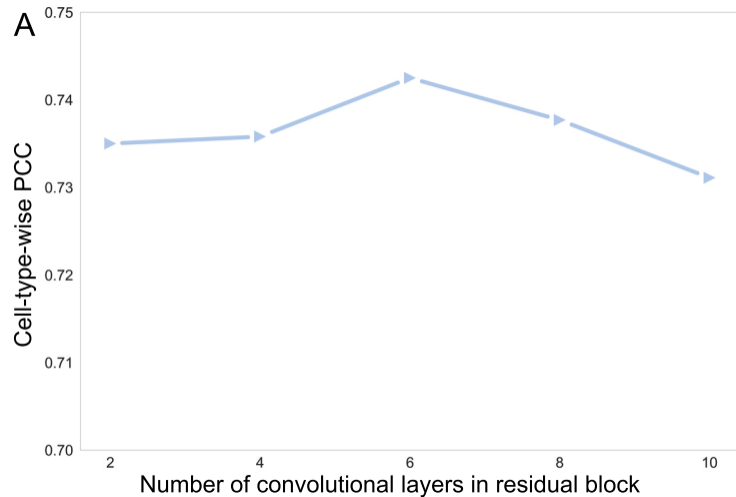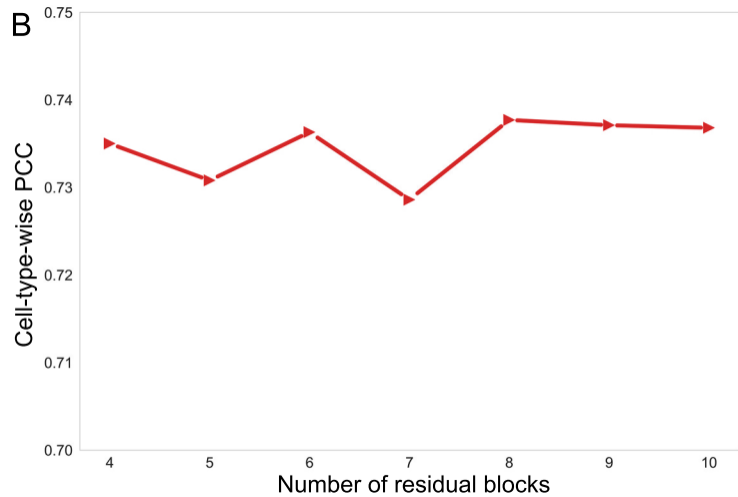

Supplement: Supplementary Figure S5 — The cross-cell-type prediction performance of ChromDragoNN with different hyperparameter settings A. The cell-type-wise PCC of ChromDragoNN with the different number of convolutional layers in each residual block. B. The cell-type-wise PCC of ChromDragoNN with the different number of residual blocks. [file mmc5.pdf]
