# Supplementary material for: DeepCAGE: Incorporating Transcription Factors in Genome-wide Prediction of Chromatin Accessibility
Source: Genomics Proteomics Bioinformatics. 2022 Mar 12;20(3):496–507. doi: 10.1016/j.gpb.2021.08.015 (PMC9801045; doi:10.1016/j.gpb.2021.08.015)
Supplement: Supplementary Figure S2 — Definition of cell type-wise and locus-wise criteria In the cell-type-wise evaluation, the auPR and Pearson’s correlation were calculated based on rows of both label matrix and predicted matrix. In the locus-wise evaluation, the Pearson’s correlation coefficient was calculated based on columns of both label matrix and predicted matrix. auPR, area under the precision-recall curve. [file mmc2.pdf]

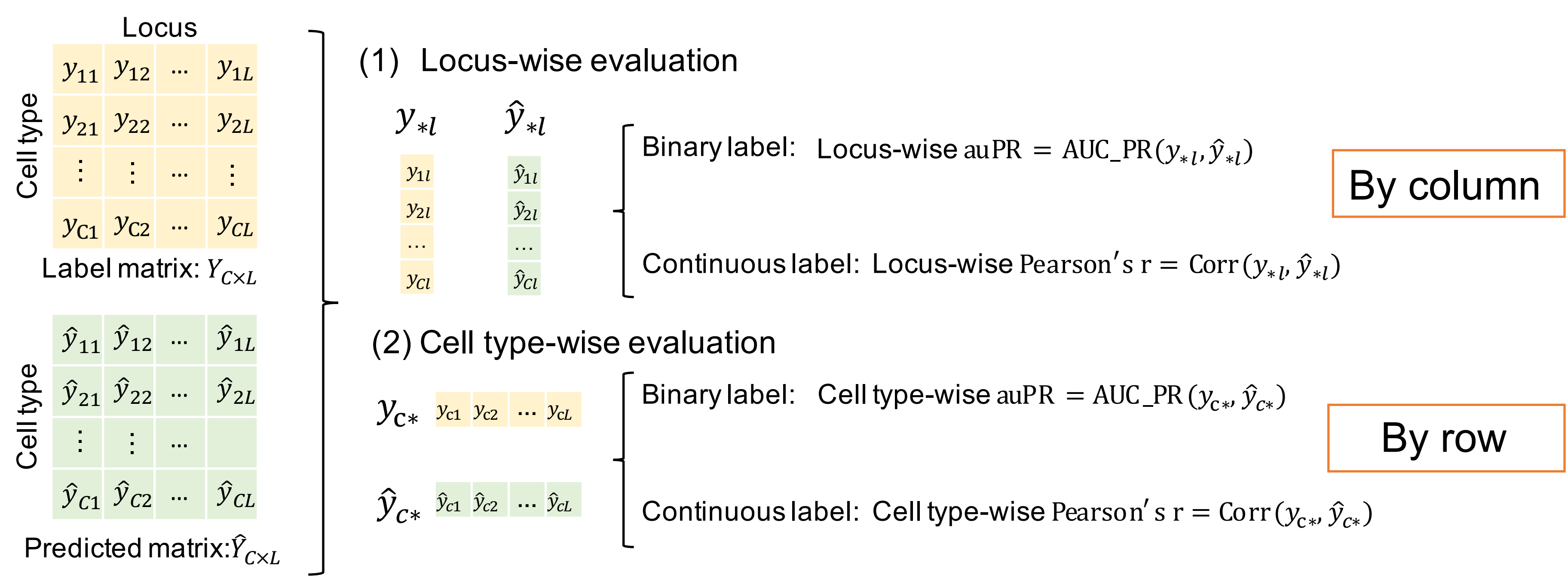

Locus
Cell type
Label matrix: $Y_{C\times L}$
Cell type
Predicted matrix: $\hat{Y}_{C\times L}$
(1) Locus-wise evaluation
y_{*l} $\hat{y}_{*l}$
Binary label: Locus-wise auPR = AUC_PR($y_{*l}, \hat{y}_{*l}$)
Continuous label: Locus-wise Pearson's r = Corr($y_{*l}, \hat{y}_{*l}$)
By column
(2) Cell type-wise evaluation
y_{c*} $\hat{y}_{c*}$
Binary label: Cell type-wise auPR = AUC_PR($y_{c*}, \hat{y}_{c*}$)
Continuous label: Cell type-wise Pearson's r = Corr($y_{c*}, \hat{y}_{c*}$)
By row
