# Supplementary material for: DeepCAGE: Incorporating Transcription Factors in Genome-wide Prediction of Chromatin Accessibility
Source: Genomics Proteomics Bioinformatics. 2022 Mar 12;20(3):496–507. doi: 10.1016/j.gpb.2021.08.015 (PMC9801045; doi:10.1016/j.gpb.2021.08.015)
Supplement: Supplementary Figure S3 — Model ablation analysis of DeepCAGE A. Four models were designed by considering different inputs. B. By removing both expression and motif scores of transcription factors (model 1), the median cell type-wise PCC decreases from 0.795 to 0.660. If only gene expression (model 2) or motif scores (model 3) are discarded, the median cell type-wise decreases to 0.664 and 0.759, respectively. PCC, Pearson correlation coefficient. [file mmc3.pdf]

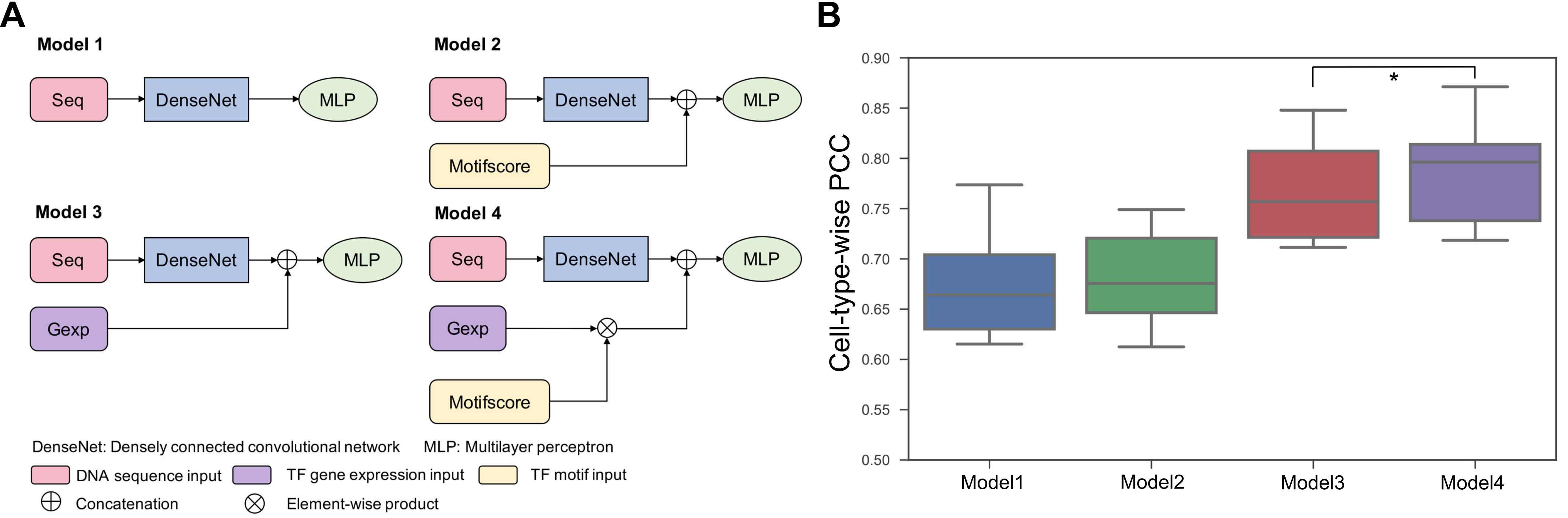
A
Model 1
Seq
DenseNet
MLP
Model 2
Seq
DenseNet
MLP
Motifscore
Model 3
Seq
DenseNet
MLP
Gexp
Model 4
Seq
DenseNet
MLP
Gexp
Motifscore
DenseNet: Densely connected convolutional network
MLP: Multilayer perceptron
DNA sequence input
TF gene expression input
TF motif input
Concatenation
Element-wise product
B
Cell-type-wise PCC
0.90
0.85
0.80
0.75
0.70
0.65
0.60
0.55
0.50
*
Model1
Model2
Model3
Model4
