# Supplementary material for: DeepCAGE: Incorporating Transcription Factors in Genome-wide Prediction of Chromatin Accessibility
Source: Genomics Proteomics Bioinformatics. 2022 Mar 12;20(3):496–507. doi: 10.1016/j.gpb.2021.08.015 (PMC9801045; doi:10.1016/j.gpb.2021.08.015)
Supplement: Supplementary Figure S4 — Ablation study for model architecture of DeepCAGE A. We implemented DeepCAGE model with two different architectures (DenseNet and ResNet). Note that we used a ResNet with three layers (equal to the number of dense blocks) and the number of hidden nodes and the convolutional kernel size is the same as convolutional layers in DenseNet. B. DeepCAGE with DenseNet architecture achieves a median cell-type-wise PCC of 0.795, while DeepCAGE with ResNet architecture achieves an average cell-type-wise PCC of 0.731. [file mmc4.pdf]

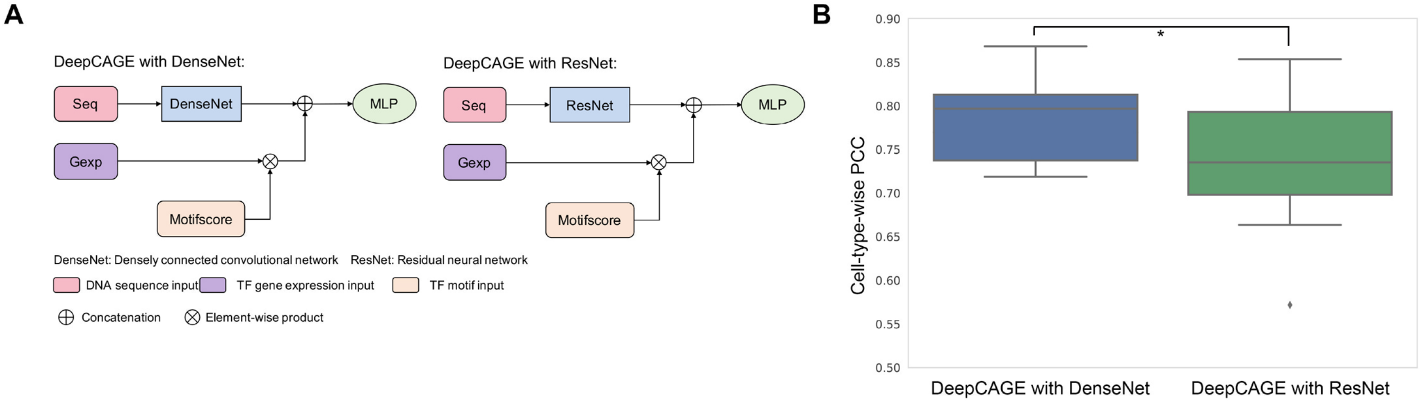

A
DeepCAGE with DenseNet:
Seq
DenseNet
MLP
Gexp
Motifscore
DeepCAGE with ResNet:
Seq
ResNet
MLP
Gexp
Motifscore
DenseNet: Densely connected convolutional network
ResNet: Residual neural network
DNA sequence input
TF gene expression input
TF motif input
Concatenation
Element-wise product
B
Cell-type-wise PCC
0.90
0.85
0.80
0.75
0.70
0.65
0.60
0.55
0.50
*
DeepCAGE with DenseNet
DeepCAGE with ResNet
