# Supplementary material for: DeepCAGE: Incorporating Transcription Factors in Genome-wide Prediction of Chromatin Accessibility
Source: Genomics Proteomics Bioinformatics. 2022 Mar 12;20(3):496–507. doi: 10.1016/j.gpb.2021.08.015 (PMC9801045; doi:10.1016/j.gpb.2021.08.015)
Supplement: Supplementary Table S1 — Hyperparameters of the DeepCAGE model Note: The hyperparameters were determined by mainly focusing on choosing the number of dense blocks {1,3,5}, learning rate {0.01,0.001,0.0001}, number of hidden nodes in the feed-forward network {128,256,512} with the help of the Hyperopy library (http://hyperopt.github.io/hyperopt/) [file mmc6.docx]

**Table S1** **Hyperparameters of the DeepCAGE model**.

| **Layers** | **Output shape** | **Operation** |
| --- | --- | --- |
| Input | $4\times1000\times1$ |  |
| Convolution | $1\times986\times160$ | $4\times15$ conv, stride=1 |
|  |  | Batch normalization |
| Pooling | $1\times246\times160$ | $1\times4$ max pooling |
|  |  | 20% dropout |
| Dense Block (1) | $1\times246\times320$ | $\left( \begin{matrix} 1\times1 \mathrm{conv} \\ 1\times3 \mathrm{conv} \end{matrix} \right)\times5$ |
| Convolution | $1\times235\times160$ | $1\times12$ conv, stride=1 |
|  |  | Batch normalization |
| Pooling | $1\times58\times160$ | $1\times4$ max pooling |
|  |  | 20% dropout |
| Dense Block (2) | $1\times58\times320$ | $\left( \begin{matrix} 1\times1 \mathrm{conv} \\ 1\times3 \mathrm{conv} \end{matrix} \right)\times5$ |
| Convolution | $1\times47\times160$ | $1\times12$ conv, stride=1 |
|  |  | Batch normalization |
| Pooling | $1\times11\times160$ | $1\times4$ max pooling |
|  |  | 20% dropout |
| Dense Block (3) | $1\times11\times320$ | $\left( \begin{matrix} 1\times1 \mathrm{conv} \\ 1\times3 \mathrm{conv} \end{matrix} \right)\times5$ |
| Flatten | 3520 | Reshape |
| Hybrid layer | 3922 | Concatenated with TF features |
| Classification/Regression layer | 1 | 512 fully-connected |
|  |  | Batch normalization+50% dropout |
|  |  | Softmax/Linear |

*Note*: The hyperparameters were determined by mainly focusing on choosing the number of dense blocks {1,3,5}, learning rate {0.01,0.001,0.0001}, number of hidden nodes in the feed-forward network {128,256,512} with the help of the Hyperopy library (http://hyperopt.github.io/hyperopt/).
