# Supplementary material for: DeepCAGE: Incorporating Transcription Factors in Genome-wide Prediction of Chromatin Accessibility
Source: Genomics Proteomics Bioinformatics. 2022 Mar 12;20(3):496–507. doi: 10.1016/j.gpb.2021.08.015 (PMC9801045; doi:10.1016/j.gpb.2021.08.015)
Supplement: Supplementary Table S6 — Information of the 55 cell types used in this study [file mmc11.docx]

**Table S6** **Information of the 55 cell types used in this study**.

| **Cell type ID and cell type name** | | | | | | | | | |
| --- | --- | --- | --- | --- | --- | --- | --- | --- | --- |
| 1 | A549 | 12 | Esophagus muscularis mucosa | 23 | Heart left ventricle | 34 | Mammary epithelial cell | 45 | Sigmoid colon |
| 2 | Adrenal gland | 13 | Esophagus squamous epithelium | 24 | HeLa-S3 | 35 | MCF-7 | 46 | Skeletal muscle myoblast |
| 3 | Ascending aorta | 14 | Fibroblast of dermis | 25 | Hepatocyte | 36 | Myotube | 47 | SK-N-SH |
| 4 | Astrocyte | 15 | Fibroblast of lung | 26 | HepG2 | 37 | OCI-LY7 | 48 | Smooth muscle cell |
| 5 | BE2C | 16 | Fibroblast of skin of abdomen | 27 | IMR-90 | 38 | Omental fat pad | 49 | Spleen |
| 6 | Bipolar neuron | 17 | Fibroblast of the aortic adventitia | 28 | Ishikawa | 39 | Osteoblast | 50 | T47D |
| 7 | Breast epithelium | 18 | Foreskin fibroblast | 29 | K562 | 40 | Panc1 | 51 | Thyroid gland |
| 8 | Bronchial epithelial cell | 19 | Gastrocnemius medialis | 30 | Keratinocyte | 41 | Pancreas | 52 | Tibial nerve |
| 9 | CD14-positive monocyte | 20 | GM12878 | 31 | Kidney epithelial cell | 42 | Peyer's patch | 53 | Transverse colon |
| 10 | Endothelial cell of umbilical vein | 21 | GM23338 | 32 | Lower leg skin | 43 | Prostate gland | 54 | Uterus |
| 11 | Epithelial cell of proximal tubule | 22 | H1-hESC | 33 | Lung | 44 | Right lobe of liver | 55 | Vagina |
