# Supplementary material for: DeepCAGE: Incorporating Transcription Factors in Genome-wide Prediction of Chromatin Accessibility
Source: Genomics Proteomics Bioinformatics. 2022 Mar 12;20(3):496–507. doi: 10.1016/j.gpb.2021.08.015 (PMC9801045; doi:10.1016/j.gpb.2021.08.015)
Supplement: Supplementary Table S7 — Data partition in the five-fold cross-validation experiment [file mmc12.docx]

**Table S7** **Data partition in the five-fold cross-validation experiment**

| **Fold index** | **Training cell-type IDs** | **Test cell-type IDs** |
| --- | --- | --- |
| 1 | 12 13 14 15 16 17 18 19 20 21 22 23 24 25 26 27 28 29 30 31 32 33 34 35 36 37 38 39 40 41 42 43 44 45 46 47 48 49 50 51 52 53 54 55 | 1 2 3 4 5 6 7 8 9 10 11 |
| 2 | 1 2 3 4 5 6 7 8 9 10 11 23 24 25 26 27 28 29 30 31 32 33 34 35 36 37 38 39 40 41 42 43 44 45 46 47 48 49 50 51 52 53 54 55 | 12 13 14 15 16 17 18 19 20 21 22 |
| 3 | 1 2 3 4 5 6 7 8 9 10 11 12 13 14 15 16 17 18 19 20 21 22 34 35 36 37 38 39 40 41 42 43 44 45 46 47 48 49 50 51 52 53 54 55 | 23 24 25 26 27 28 29 30 31 32 33 |
| 4 | 1 2 3 4 5 6 7 8 9 10 11 12 13 14 15 16 17 18 19 20 21 22 23 24 25 26 27 28 29 30 31 32 33 45 46 47 48 49 50 51 52 53 54 55 | 34 35 36 37 38 39 40 41 42 43 44 |
| 5 | 1 2 3 4 5 6 7 8 9 10 11 12 13 14 15 16 17 18 19 20 21 22 23 24 25 26 27 28 29 30 31 32 33 34 35 36 37 38 39 40 41 42 43 44 | 45 46 47 48 49 50 51 52 53 54 55 |

*Note*: Each fold contains 44 training cell types and 11 test cell types.
